# A real life evaluation of non invasive ventilation in acute cardiogenic pulmonary edema: a multicenter, perspective, observational study for the ACPE SIMEU study group

**DOI:** 10.1186/s12873-018-0216-z

**Published:** 2018-12-29

**Authors:** Stefano Aliberti, Valentina Diana Rosti, Chiara Travierso, Anna Maria Brambilla, Federico Piffer, Giuseppina Petrelli, Chiara Minelli, Daniele Camisa, Antonio Voza, Giovanna Guiotto, Roberto Cosentini, on behalf of the SIMEU ACPE study group, Andrea Cellini, Tommaso Maraffi, Caterina Bonino, Paolo Groff, Andrea Bellone, Massimo Giorgino, Lucia Morelli, Federico Furlan, Antonio Villa, Andrea Purro, Federica Molinaro, Adolfo Di Nardo, Raffaella Francesconi, Fabio Guzzin, Marta Salzillo, Vitaliano Tiscione

**Affiliations:** 1 Department of Pathophysiology and Transplantation, University of Milan, Cardio-Thoracic Unit and Adult Cystic Fibrosis Center, Fondazione IRCCS Ca, Granda Ospedale Maggiore Policlinico, Milan, Italy; 2 Emergency Medicine Department, ASST Papa Giovanni XIII, Bergamo, Italy; 3 Respiratory Unit, ASST Rhodense Ospedale Salvini, Viale Forlanini 95, 20024 Garbagnate Milanese, Italy; 4 Emergency Department, IRCCS Fondazione Ospedale Maggiore Policlinico Ca’ Granda, Milan, Italy; 5 Department of Pulmonology, Hospital of Arco, APSS, Trento, Italy; 6 Emergency Department, Presidio Ospedaliero Madonna del Soccorso, San Benedetto del Tronto, Ascoli Piceno, Italy; 7 Emergency Department, Valduce Hospital, Como, Italy; 8 Emergency Department, Vizzolo Predabissi Hospital, AO Melegnano, Milan, Italy; 9 Emergency Department, IRCCS Istituto Clinico Humanitas, Rozzano, Milan, Italy; 10 Emergency Department, San Paolo Hospital, Naples, Italy

**Keywords:** Non-invasive ventilation, Respiratory, Heart failure, Emergency department

## Abstract

**Background:**

During the past three decades conflicting evidences have been published on the use of non-invasive ventilation (NIV) in patients with acute cardiogenic pulmonary edema (ACPE). The aim of this study is to describe the management of acute respiratory failure (ARF) due to ACPE in twelve Italian emergency departments (EDs). We evaluated prevalence, characteristics and outcomes of ACPE patients treated with oxygen therapy, continuous positive airway pressure (CPAP) or Bi-level positive airway pressure (BiPAP) on admission to the EDs.

**Methods:**

In this multicenter, prospective, observational study, consecutive adult patients with ACPE were enrolled in 12 EDs in Italy from May 2009 to December 2013. Three study groups were identified according to the initial respiratory treatment: patients receiving oxygen therapy, those treated with CPAP and those treated with BiPAP. Treatment failure was evaluated as study outcome.

**Results:**

We enrolled 1293 patients with acute cardiogenic pulmonary edema. 273 (21%) began with oxygen, 788 (61%) with CPAP and 232 (18%) with BiPAP. One out of four patient who began with oxygen was subsequently switched to NIV and initial treatment with oxygen therapy had an odds ratio for treatment failure of 3.65 (95% CI: 2.55–5.23, *p* < 0.001). Conclusions: NIV seems to be the first choice for treatment of ARF due to ACPE, showing high clinical effectiveness and representing a rescue option for patients not improving with conventional oxygen therapy.

## Background

Acute cardiogenic pulmonary edema (ACPE) represents a heterogeneous [1–3] syndrome with a mortality rate up to 9.5% [4]. Although many clinical trials by the end of ‘90s showed that noninvasive ventilation (NIV) decreases the need for intubation and mortality in ACPE [5], a study by Gray and colleagues cast doubt on previous published data [6]. From one hand, medical therapy of ACPE is well defined in clinical practice, following strong recommendations suggested by international guidelines on the use of diuretics, opiates, vasodilators, vasopressors and thromboembolism prophylaxis [4]. On the other hand, treatment of acute respiratory failure (ARF), beyond oxygen administration, remains a question of controversy. Most of the recent guidelines do not provide a strong recommendation on the use of NIV to treat ARF due to ACPE [4, 7–10]. In view of the absence of a strong recommendation on the use of continuous positive airway pressure (CPAP) or bi-level positive airway pressure (BiPAP) to treat ARF due to ACPE, an evaluation on the use of both techniques in daily clinical practice is needed. The aim of this study is to describe the management of ARF due to ACPE in twelve Italian emergency departments. We evaluated prevalence, characteristics and outcomes of ACPE patients treated with oxygen therapy, CPAP or BiPAP on admission according to attending physicians judgement.

## Methods

This was a multicenter, prospective, observational study on consecutive adult patients admitted with diagnosis of ACPE to 12 EDs in Italy from May 2009 to December 2013, see Appendix. The Institutional Review Boards of all the hospitals approved the study, while the informed consent was waived due to the observational nature of the study. ACPE was defined by the presence of all the following: acute-onset dyspnea, widespread pulmonary rales and pulmonary congestion on chest X-ray (CXR), plus at least one among: 1) respiratory distress; 2) respiratory rate ≥ 30 breath/minute; 3) pH < 7.35 and partial pressure of carbon dioxide on arterial blood > 45 mmHg in Venturi Mask with an inspiratory fraction of oxygen of 0.50 (flow of 12 L/min). Patients who required an immediate endotracheal intubation or other life-saving interventions were excluded from the study. Data recorded on admission to the EDs included demographics, comorbidities, clinical and instrumental data (vital signs, arterial blood gas analysis, blood chemistry, electrocardiogram, CXR), medical therapy, respiratory support and complications. All data were collected on an electronic case report form and anonymously stored in a centralized database (www.acpe.it). Three study groups were identified according to the initial respiratory treatment: patients receiving oxygen therapy, those treated with CPAP and those treated with BiPAP. All the options were available in the twelve participating centers and the decision to initiate oxygen, CPAP or BiPAP was taken by the attending physicians, based on their personal judgement and local attitude. The primary study outcome was treatment failure defined as at least one of the following: 1) discontinuation of the technique (either oxygen or CPAP or BiPAP) after one hour from its initiation for a.

deterioration of either gas exchange or clinical status; 2) death. All statistical analyses were run using SPSS®, version 20, for MAC platform. Continuous data were presented as median values with interquartile range (IQR) of the 25th and 75th percentiles. Categorical data were presented as absolute number and percentage. According to previous literature, the sample size calculation was based on an assumed rate of treatment failure of 25% in the oxygen therapy group and of 15% in the group of patients treated with either CPAP or BiPAP. If these assumptions were correct, a minimum of 500 patients would be enrolled to detect such a reduction with a power of 90% and a two-sided type 1 error rate of 5%. Data among groups were compared using a Mann-Whitney or Kruskal-Wallis tests if continuous, and a chi-squared test or Fisher exact test if categorical. A *p*-value of < 0.05 was considered statistically significant.

## Results

A total of 1300 patients were enrolled in the study. 7 patients were excluded due to their immediate intubation. Demographics, comorbidities and data on hospital admission of the final population of 1293 patients (median age: 81 years, 51% males) are reported in Table 1. The five most frequent causes of ACPE included hypertensive crisis (22%), acute coronary syndrome (19%), cardiac arrhythmia (8%), low respiratory tract infections (8%), valvular disease or ruptured mitralic *chordae* (3%).

**Table 1:** Demographics, comorbidities, clinical and laboratory data on hospital admission of the study population, according to the three study groups

	Study population	Oxygen Group *n* = 273	CPAP Group *n* = 788	BiPAP Group *n* = 232	p^+^	p^#^	p^§^
n. (%)	1293 (100)	273 (100)	788 (100)	232 (100)			
Demographics, n.(%)							
Age, median (IQR) years	81 (73–86)	80 (73–85)	81 (73–86)	80 (74–86)	0.366	0.828	0.369
Female sex	635 (49)	137 (50)	389 (49)	107 (46)	0.548	0.385	0.648
Comorbidities, n(%)							
COPD	406 (31)	92 (34)	241 (31)	72 (32)	0.633	0.798	0.357
Diabetes	495 (38)	112 (41)	292 (37)	91 (39)	0.482	0.549	0.294
Chronic kidney disease	353 (27)	87 (32)	215 (27)	51 (22)	0.046	0.106	0.057
Hypertension	974 (75)	198 (72)	594 (75)	182 (78)	0.306	0.336	0.227
Atrial fibrillation	264 (20)	64 (23)	154 (20)	46 (20)	0.376	0.924	0.163
Congestive heart failure	315 (24)	67 (24)	178 (23)	70 (30)	0.061	0.018	0.938
Coronary artery disease	599 (46)	134 (49)	360 (46)	105 (45)	0.585	0.909	0.304
Valvulopaty	300 (23)	69 (25)	193 (24)	38 (16)	0.024	0.009	0.361
ACPE in the previous 12 months	295 (23)	63 (23)	178 (23)	54 (23)	0.970	0.826	0.908
Data on admission, n.(%)							
Systolic blood pressure, median (IQR) mmHg	160 (140–190)	159 (135–180)	164 (140–190)	162 (140–185)	0.043	0.900	0.012
Diastolic blood pressure, median (IQR) mmHg	90 (80–105)	90 (75–100)	95 (80–107)	87 (75–100)	0.000	0.001	0.025
Mean blood pressure, median (IQR) mmHg	115 (98–131)	113 (95–127)	117 (99–133)	113 (98–127)	0.084	0.217	0.064
Hypotension*	19 (1)	4 (1)	14 (2)	1 (< 1)	0.321	0.132	0.992
Heart rate, median (IQR) beats/min	104 (90–120)	100 (86–113)	108 (95–120)	102 (92–120)	0.000	0.102	0.000
Respiratory rate, median (IQR) breaths/min	34 (30–40)	30 (26–36)	35 (30–40)	35 (30–40)	0.000	0.824	0.000
Respiratory rate ε breaths/min, n. (%)	568 (49)	72 (29)	374 (55)	122 (53)	0.000	0.620	0.000
pH, median (IQR)	7.29 (7.20–7.37)	7.37 (7.28–7.42)	7.28 (7.20–7.35)	7.23 (7.13–7.31)	0.000	0.000	0.000
pH < 7.35, n. (%)	862 (69)	115 (44)	550 (72)	197 (87)	0.000	0.000	0.000
PaCO_2_, median (IQR) mmHg	47 (38–59)	42 (35–51)	47 (39–57)	60 (45–72)	0.000	0.000	0.000
PaCO2 ≤ 35 mmHg, n. (%)	235 (18)	74 (27)	136 (17)	25 (11)	0.000	0.012	0.000
PaCO2 ≥ 45 mmHg, n. (%)	732 (58)	116 (43)	440 (57)	176 (76)	0.000	0.000	0.000
PaO_2_/FiO_2_ ratio, median (IQR)	195 (134–247)	209 (155–252)	190 (128–248)	181 (136–243)	0.119	0.623	0.056
PaO2/FiO2 ratio < 200, n. (%)	652 (51)	116 (43)	412 (54)	124 (54)	0.006	0.992	0.001
HCO_3_^−^, median (IQR) mmol/L	22 (20–26)	23 (20–26)	22 (19–25)	23 (20–26)	0.004	0.042	0.009
HCO_3_^−^ > 24 mEq/L	415 (33)	104 (38)	226 (30)	85 (37)	0.011	0.045	0.025
Lactates, median (IQR) mmol/L	2.2 (1.3–3.9)	2 (1.1–3.1)	2.3 (1.4–4)	2.25 (1.2–4)	0.070	0.573	0.026
Kelly scale > 3	61 (5)	10 (4)	27 (3)	24 (11)	0.000	0.000	0.452

Footnotes: *CPAP* continuous positive airway pressure, *BiPAP* Bi-level Positive Airway Pressure, *IQR* 25–75 interquartile range, *COPD* chronic obstructive pulmonary disease, *ACPE* acute cardiogenic pulmonary edema, *PaO*_*2*_ partial pressure of oxygen in the arterial blood, *FiO*_*2*_ inspiratory fraction of oxygen, *PaCO*_*2*_ partial pressure of carbon dioxide in the arterial blood, *HCO*_*3*_^−^ bicarbonates, *Hypothension was defined as systolic blood pressure < 90 mmHg; ^+^among the three groups; ^#^between CPAP Group vs. BiPAP Group; ^§^between Oxygen Group vs. CPAP *plus* BiPAP Groups

**Fig. 1:**
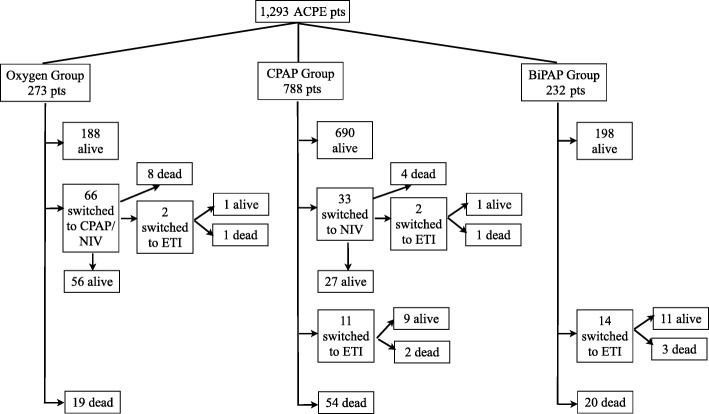
Flow chart of the study population. Pts. = patients; CPAP = continuous positive airway pressure; BiPAP: Bi-level Positive Airway Pressure; ETI = endotracheal intubation

ARF was treated as follows: oxygen therapy for 273 patients (21%), CPAP for 788 (61%) and BiPAP for 232 (18%), see Fig. 1. Among patients who were treated with either CPAP or BiPAP anytime during the hospitalization the following complications were detected: pneumothorax in one patient, vomiting in 15 patients and shock in 25 patients.

**Fig. 2:**
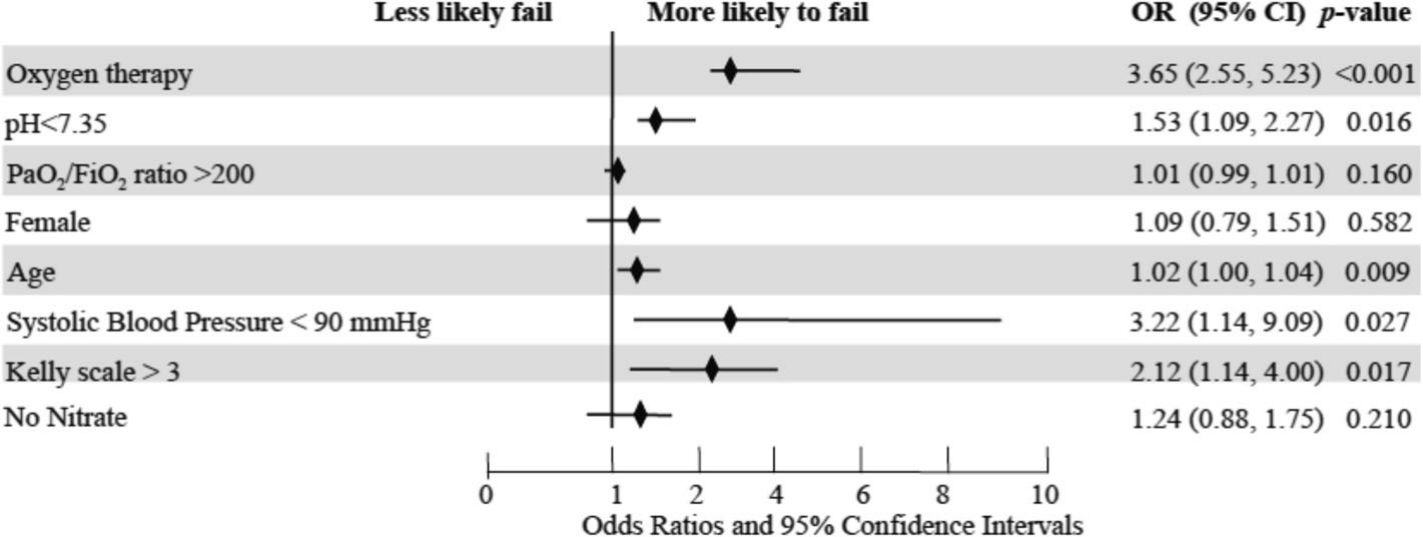
Independent predictors for clinical failure in the study population. OR: Odds ratio; CI: confidence intervals; PaO2: partial pressure of oxygen in the arterial blood; FiO2: inspiratory fraction of oxygen; no nitrates: group of patients that were not treated with nitrates

A total of 112 patients (9%) died during hospitalization, including 28 patients (10%) in the oxygen group, 61 (8%) in the CPAP group and 23 (10%) in the BiPAP group, *p* = 0.330, see Fig. 1. Early mortality (within 24 h of admission from the ED) was 3% (38 patients): 9 patients (3%) in the oxygen group, 21 (3%) in the CPAP group and 8 (3%) in the BiPAP group, *p* = 0.760. A total of 29 patients (2%) underwent endotracheal intubation (ETI) in the entire study population: 2 (1%) in the oxygen group, 13 (2%) in the CPAP group, and 14 (6%) in the BiPAP group, *p* < 0.05. Treatment failure was experienced by 85 patients (31%) in the oxygen group, 98 (12%) in the CPAP group and 34 (15%) in the BiPAP group, p < 0.05. A total of 66 patients out of 273 (24%) who began with oxygen were subsequently switched to NIV. Median length of stay was 9 days (IQR 5–13) in the entire study population and no significant differences were detected among the three study groups, *p* = 0.533. After adjustment for several confounders (including arterial pH, PaO_2_/FiO_2_ ratio, sex, age, systolic blood pressure, confusion on admission and nitrate use), an initial treatment with oxygen therapy showed an odds ratio for treatment failure of 3.65 (95% CI: 2.55–5.23, *p* < 0.001), see Fig. 2.

## Discussion

Our study shows that physicians apply NIV in almost 80% of patients presenting to the ED with ACPE. Notably, one out of four patients who were initially treated with oxygen therapy were switched to NIV. We show that an initial treatment with oxygen therapy is associated with more than 3 fold risk of treatment failure during hospitalization.

So far, no real life studies evaluated the use of NIV in patients with ACPE. The EFICA study enrolled 599 patients with acute decompensated heart failure (ADHF) and among them 82% had ACPE treated in intensive care unit, showing a total mortality of 27% at 4 weeks [1]. Previous data on mortality have been published mainly from either registries or surveys enrolling patients with ADHF, ACPE, heart failure *plus* hypertension, right heart failure and cardiogenic shock [2, 3]. We excluded 7 patients who were intubated on admission to the ED and we reported in-hospital mortality for patients with ACPE of 9%. Our mortality rate is in line with that published by both the EHFS2 study group and Gray and coworkers [3, 6].

Our study points out a diffuse perception among physicians that NIV is more beneficial than oxygen to manage ARF in ACPE patients. We found that emergency physicians apply in clinical practice either CPAP or BiPAP in almost 80% of ACPE patients and the vast majority of them are initially treated with CPAP. These data are interesting in light of the heterogeneity of the recommendations suggested by international societies and findings published by Grey and coworkers [3, 8, 9]. Furthermore, the fact that in our study 21% of ACPE patients were treated with standard oxygen therapy seems even more interesting; this choice could be attributed to personal or local attitude and surely it is partially justified by a less severe presentation of these patients; nevertheless, the switch to NIV of one out of four patients seems to suggest an initial under-treatment despite many guidelines does not recommend NIV so firmly. In line with our results, the last ERS/ATS guidelines recommend NIV for ARF due to ACPE more strongly (10). Finally, our results are of special interest in view of the limited use of NIV in the emergency room as recently pointed out [11]. One possible explanation of the large use of NIV/CPAP in our study may be found in the high severity of the disease on admission, since two thirds of our patients were acidotic on admission. According to our results, emergency physicians seem to be more willing to use NIV in case of high respiratory rate, low pH and high level of PaCO2. Furthermore, main drivers for choosing BiPAP (in comparison to CPAP) were a Kelly score greater than 3, a low pH and a high PaCO2. Due to its prospective nature, our study lacks strict criteria to initiate oxygen, CPAP or BiPAP, nevertheless we aimed to describe the real life management of ARF in the Italians EDs. Only a randomized controlled trial without crossover between groups could detect a potential difference in mortality between oxygen and NIV, but we think that the rate of switch from oxygen to NIV in our study should lead physicians to monitor more carefully ACPE patients treated with oxygen therapy. Other limitations of our study are that we were not able to evaluate medium and long-term outcomes and the following episodes of ACPE and re-hospitalizations. This study is strengthened by the multicentric and real life design, enrolling consecutive patients affected by ACPE (according to a strict definition) and specifically focused on NIV. A large RCT comparing these three methods is needed and we suggest clinical failure being the primary endpoint without patients’ crossover among groups. In conclusion, NIV seems to be the first choice for treatment of ARF due to ACPE, showing high clinical effectiveness and representing a rescue option for patients not improving on conventional oxygen therapy.

## Abbreviations

ACPE: acute cardiogenic pulmonary edema
ADHF: acute decompensated heart failure
ARF: acute respiratory failure
BiPAP: bi-level positive airway pressure
CI: confidence interval
COPD: chronic obstructive pulmonary disease
CPAP: continuous positive airway pressure
CXR: chest X-ray
ED: emergency department (ED)
ETI: endotracheal intubation
FiO_2_: inspiratory fraction of oxygen
HCO_3_: bicarbonates
IQR: interquartile range
NIV: non-invasive ventilation
OR: odds ratio
PaCO_2_: partial pressure of carbon dioxide in the arterial blood
PaO2: partial pressure of oxygen in the arterial blood
